# Subacute Partially Reversible Leukoencephalopathy Expands the Aicardi–Goutières Syndrome Phenotype

**DOI:** 10.3390/brainsci13081169

**Published:** 2023-08-05

**Authors:** Isabella Peixoto de Barcelos, Clarissa Bueno, Luís Filipe S. Godoy, André Pessoa, Larissa A. Costa, Fernanda C. Monti, Katiane Souza-Cabral, Clarice Listik, Diego Castro, Bruno Della-Ripa, Fernando Freua, Laís C. Pires, Lia T. Krüger, José Luiz D. Gherpelli, Flavia B. Piazzon, Fabiola P. Monteiro, Leandro T. Lucato, Fernando Kok

**Affiliations:** 1 Child Neurology Service, Department of Neurology, University of São Paulo School of Medicine, Dr. Enéas de Carvalho Aguiar, 255, 5th Floor, São Paulo 05403-000, SP, Brazil; isabellapeixotobarcelos@gmail.com (I.P.d.B.); clarissa.bueno@hc.fm.usp.br (C.B.); andrepessoa10@yahoo.com.br (A.P.); fernanda.monti@gmail.com (F.C.M.); kate_souza@yahoo.com.br (K.S.-C.); clarice.listik@gmail.com (C.L.); decastroneurologia@gmail.com (D.C.); brunodellaripa@gmail.com (B.D.-R.); fernandofreua@gmail.com (F.F.); jldg@osite.com.br (J.L.D.G.); flapiazzon@gmail.com (F.B.P.); 2 Department of Radiology, University of São Paulo School of Medicine, São Paulo 05403-000, SP, Brazil; fil.godoy@gmail.com (L.F.S.G.);; 3 Albert Sabin Children’s Hospital, Ceara State University, Fortaleza 60714-903, CE, Brazil; 4 Mendelics Genomic Analysis, São Paulo 02511-000, SP, Brazil; larissa.athayde@mendelics.com.br (L.A.C.); fabiola.monteiro@mendelics.com.br (F.P.M.); 5 Paulo Niemeyer State Institute of Brain, Rio de Janeiro 20230-024, RJ, Brazil; laispires@terra.com.br (L.C.P.); liatheophilo@hotmail.com (L.T.K.); 6 Albert Einstein Hospital, São Paulo 05652-900, SP, Brazil

**Keywords:** reversible leukoencephalopathy, Aicardi–Goutières syndrome, acute demyelinating encephalomyelitis

## Abstract

Objective: To report a series of atypical presentations of Aicardi–Goutières syndrome. Methods: Clinical, neuroimaging, and genetic data. Results: We report a series of six unrelated patients (five males) with a subacute loss of developmental milestones, pyramidal signs, and regression of communication abilities, with onset at ages ranging from 7 to 20 months, reaching a nadir after 4 to 24 weeks. A remarkable improvement of lost abilities occurred in the follow-up, and they remained with residual spasticity and dysarthria but preserved cognitive function. Immunization or febrile illness occurred before disease onset in all patients. CSF was normal in two patients, and in four, borderline or mild lymphocytosis was present. A brain CT scan disclosed a subtle basal ganglia calcification in one of six patients. Brain MRI showed asymmetric signal abnormalities of white matter with centrum semi-ovale involvement in five patients and a diffuse white matter abnormality with contrast enhancement in one. Four patients were diagnosed and treated for acute demyelinating encephalomyelitis (ADEM). Brain imaging was markedly improved with one year or more of follow-up (average of 7 years), but patients remained with residual spasticity and dysarthria without cognitive impairment. Demyelination relapse occurred in a single patient four years after the first event. Whole-exome sequencing (WES) was performed in all patients: four of them disclosed biallelic pathogenic variants in *RNASEH2B* (three homozygous p.Ala177Thr and one compound heterozygous p.Ala177Thr/p.Gln58*) and in two of them the same homozygous deleterious variants in *RNASEH2A* (p.Ala249Val). Conclusions: This report expands the phenotype of AGS to include subacute developmental regression with partial clinical and neuroimaging improvement. Those clinical features might be misdiagnosed as ADEM.

## 1. Introduction

Aicardi–Goutières syndrome (AGS) is a genetically heterogeneous disorder with onset usually in the first months of age clinically characterized by irritability, aseptic hyperthermia, loss of developmental milestones, microcephaly, spastic diplegia, dystonia, and intellectual impairment [1,2,3]. Brain neuroimaging usually discloses diffuse or frontotemporal white matter (WM) abnormalities [4], multiple calcifications, and atrophy [5]. Typically, cerebrospinal fluid shows chronic lymphocytosis with dysregulation of the type 1 interferon (1-IFN) signaling pathway [6].

In addition to the classical presentation, other neurological phenotypes have been reported, including 1. prenatal onset resembling congenital infection with basal ganglia calcification [3,7]; 2. bilateral acute striatal necrosis; 3. hereditary spastic paraplegia; and 4. cerebrovascular disease [8]. Additionally, cases with onset after the first year of life have been reported [2,7,8,9,10].

Pathogenic variants in seven genes (*TREX1* [11], *RNASEH2A* [12], *RNASEH2B* [12], *RNASEH2C* [12], *SAMHD1* [13], *ADAR1* [14], and *IFIH1* [15]) have been associated with AGS. Products of all those genes are involved in nucleic acid sensing or metabolism. Their functional compromise either induces 1-IFN production or upregulation of interferon-stimulated genes, collectively called interferonopathy [16].

In addition to the typical leukoencephalopathy and intracranial calcifications, the radiological spectrum of AGS has been widened to include *RNASEH2B*-associated delayed myelination, *TREX1* deep WM cysts, widespread calcifications involving the basal ganglia, thalami, and deep WM [17], and striatal necrosis associated with *ADAR1* [18,19].

Only a few subacute partially reversible leukoencephalopathy cases have been reported but without information about their long-term outcome [20,21]. Herein, we report a series of six patients with molecular confirmation of AGS and clinical presentation of subacute encephalopathy, evolving with clinical and radiological improvement.

## 2. Data, Methods, and Materials

### 2.1. Participants’ Recruitment, Clinical Assessment, and Follow-Up

Different physicians evaluated patients at disease onset, and due to their atypical clinical-radiological evolution, a genetic investigation by WES was performed between 2015 and 2017. All genetic data were generated at the same clinical laboratory (Mendelics Genomic Analysis, Sao Paulo, SP, Brazil). After the result, due to the atypical presentation, cases were discussed with the same reference neurogeneticist in the country (Dr. F.K.), who organized with the medical team to report this series with the parents’ and patients’ consent.

They performed brain MRI at different intervals in the follow-up and remained in a rehabilitation program, including motor and speech. Patient 5 is being treated for obsessive-compulsive disorder (OCD).

### 2.2. Neuroimaging

Head CT scans were performed in all patients at least once. A brain MRI was performed on at least two occasions, first at disease onset and then when the patients started to recover milestones (at least partially). Follow-up image was available after the first event for all patients in different timeframes (mean time 17 months, range 4–58 months). Two experienced neuroradiologists reviewed all neuroimaging data (LFSG, with 10 years of experience in neuroradiology, and LTL, with 22 years).

Brain MRI was performed using 1.5 T. The protocol included axial T1-, T2-weighted and fluid-attenuated inversion recovery (FLAIR)-images, gradient-echo images (T2* or susceptibility-weighted images SWI), and diffusion-weighted images (DWI). MRI of the spinal cord included sagittal and axial T1- and T2-weighted images of the entire spine in four patients.

### 2.3. Laboratory

Except for patient 5, whose investigation was performed 20 years ago and was not available for revision, patients were submitted to an extensive laboratory and metabolic investigation, which included routine blood examination and measurement of C-reactive protein, erythrocyte sedimentation rate, creatine kinase, blood gas analysis, immunoglobulin levels, complement, quantitative plasma amino acids, ammonium, serology for hepatitis A, B and C, HIV, influenza, enterovirus, rubella, measles, toxoplasmosis, cytomegalovirus, herpesvirus, and Mycoplasma pneumonia. Cerebrospinal fluid (CSF) analysis was performed in all patients during the acute phase, but interferon levels were not measured.

### 2.4. Molecular Analysis

WES was performed after informed consent using the same procedure in all six patients. Genomic DNA was extracted from peripheral blood leukocytes or oral swabs using standard protocols. Exome sequencing, assembly, genotyping, and annotation were performed at Mendelics Genomic Analysis (Sao Paulo, SP, Brazil). Exome capture was performed using the Agilent Sure Select Clinical Research Exon V2 (Agilent Technologies, Santa Clara, CA, USA) or the Illumina Nextera Rapid Capture Exome kit (Illumina, Inc., San Diego, CA, USA). Sequencing was performed in an Illumina HiSeq 2500 or 4000 platform. Sequence reads aligned with the reference human genome (UCSC Genome Browser GrCh37/hg19) with the BWA (Burrows-Weeler Aligner) software (2009). Genotyping was performed using the Genome Analysis Toolkit (GATK). Annotation, filtering, and variant prioritization were performed using Mendelics proprietary software, allowing an in silico reduction in candidates for further analysis.

### 2.5. Standard Protocol Approvals, Registrations, and Patient Consents

The responsible doctor for each patient was contacted, and they were invited for publication. Written informed consent was obtained from all participants (or guardians of participants) in this study. The authorization has been accepted for the disclosure (consent-to-disclose) of neuroimages.

### 2.6. Data Availability Statement

All data from this study have been recorded within this article.

## 3. Results

We present a series of six unrelated patients with an unremarkable gestational and postnatal history who had previous normal neurologic development before presenting with a subacute motor and language regression episode between 7 and 20 months of age. A family history of a similar neurologic disease was absent, and parental consanguinity was present in a single patient. All patients’ cranial circumferences were normal, and they never had chilblains.

The onset of symptoms was related to febrile illness in three patients and immunization in the other three (Table 1).

All patients had typical developmental milestones expected for an age before disease onset (head control, sitting without support, craw, independent walking, and language) that were lost as it progressed. They became unable to support the head and initially were hypotonic and later spastic. The disease evolved with a subacute progressive neurologic impairment evolving to tetraparesis, reaching a nadir after one to six months of the first symptoms (Table 1). No dysphagia, respiratory failure, or consciousness impairment occurred. One patient had a symptomatic relapse four years after the first event.

At disease onset, the CSF cell count was normal (<2/mm^3^) in two of the six patients. Two had borderline pleocytosis (5 cells/mm^3^), and another had mild pleocytosis (8 and 21/mm^3^). One subject had a clinical and radiological relapse at age 5, accompanied by CSF pleocytosis (10/mm^3^).

**Figure 1:**
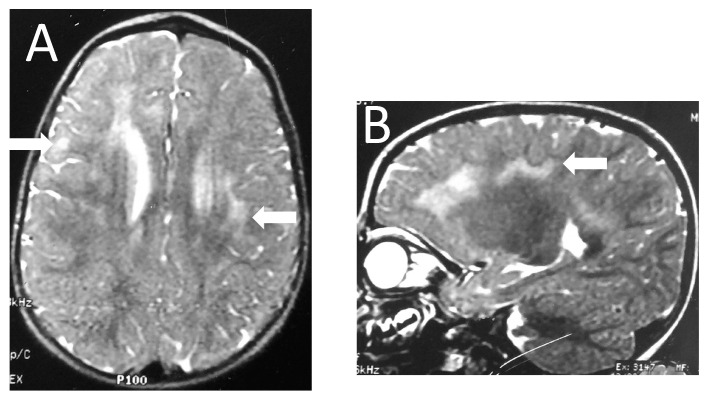
Patient 5. Axial (**A**) and sagittal (**B**) T2-weighted images show frontotemporal and parietal foci of high signal intensity (arrows) with patchy and asymmetric distribution.

**Figure 2:**
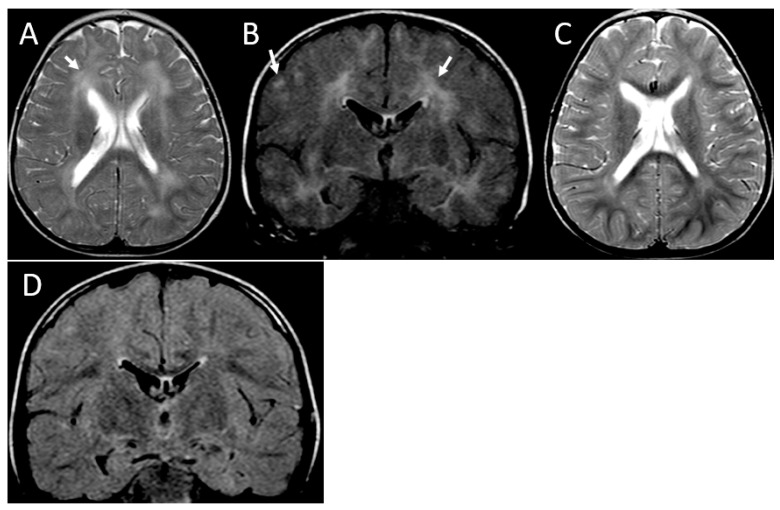
MRI of partially reversible leukoencephalopathy. Patient 3. Initial MRI at the age of 16 months, axial T2-weighted (**A**) and coronal FLAIR (**B**) images demonstrate bilateral asymmetric hyperintense areas involving subcortical and periventricular white matter (arrows). Follow-up MRI at the age of 26 months, axial T2-weighted (**C**), and coronal FLAIR (**D**) images showing improvement of the abnormal areas.

**Figure 3:**
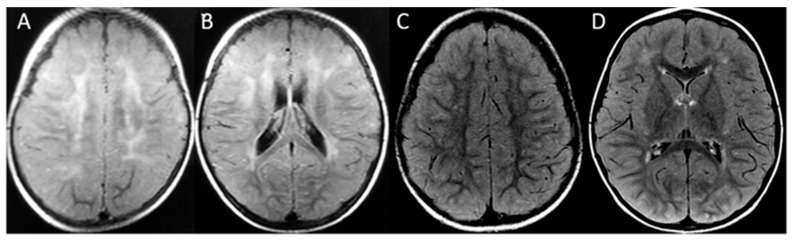
Patient 2. First MRI at 14 months old, axial FLAIR (**A**,**B**) demonstrates the frontoparietal patchy and asymmetric distribution of signal hyperintensity at the cerebral white matter. The second MRI at age 4 (**C**,**D**) shows a marked reduction in leukopathy. No atrophy is seen at any time.

**Figure 4:**
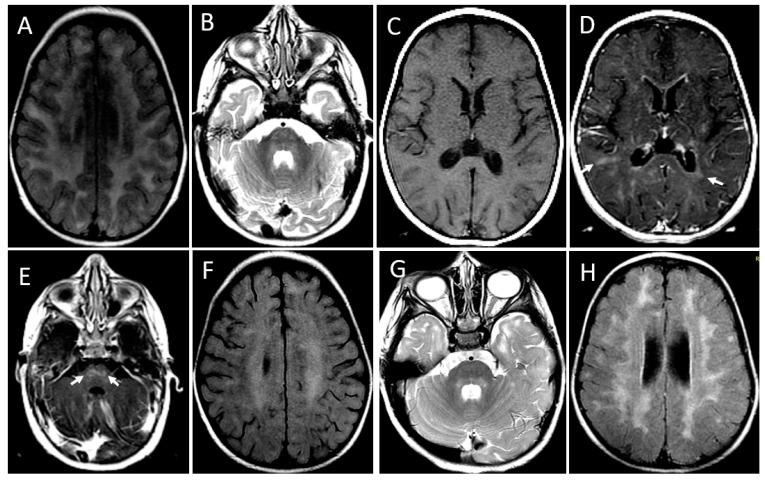
MRI showing leukoencephalopathy with contrast enhancement and a relapse-remitting course of the disease. Patient 1. Initial MRI at the age of 20 months. Axial FLAIR image (**A**) shows bilaterally confluent hyperintensity involving periventricular and subcortical white matter. Axial T2-weighted image (**B**) demonstrates hyperintensity foci in the pons (arrows). Axial T1-weighted images before (**C**) and after (**D**,**E**) gadolinium administration show scattered foci of contrast enhancement at subcortical and periventricular white matter (arrows in (**D**)) and pons (arrows in (**E**)). There is also periventricular ependymal enhancement (arrowheads in (**E**)). MRI at the age of 28 months, axial FLAIR, and T2 images (**F**,**G**) demonstrate a decrease in intensity and extension of the diffuse leukoencephalopathy (**F**) and of the signal changes in pons (**G**). In a new MRI at 5 years and 8 months, the axial FLAIR image (**H**) discloses the confluence of the hyperintense areas in both cerebral hemispheres. Contrast enhancement was seen only in the first MRI.

**Figure 5:**
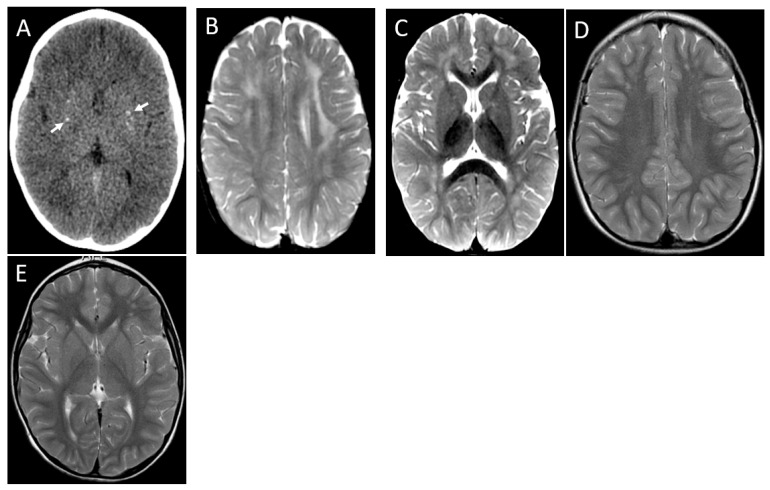
Patient 4. Axial CT image (**A**) demonstrates small foci of bilateral basal ganglia calcification (arrows). First MRI images at 17 months, axial T2 (**B**,**C**) showing bilateral frontoparietal asymmetric areas of signal hyperintensity. The second MRI at 3 years shows near complete resolution of leukopathy (**D**,**E**).

Brain MRI performed at the onset of the symptoms exhibited WM lesions in all patients, with patchy asymmetric T2/FLAIR hyperintense lesions involving subcortical and periventricular areas in five patients (Figure 1, Figure 2, Figure 3, Figure 4 and Figure 5) and diffuse WM lesions in one subject (Figure 4) (Table 2).

Contrast enhancement (periventricular region) was seen in one patient (Figure 4D,E), and pontine involvement was observed in two patients (Figure 4B,E). Head CT revealed very subtle basal ganglia calcification in a single patient (Figure 5A).

Four individuals were diagnosed at the onset of the disease as acute demyelinating encephalomyelitis (ADEM) and treated with intravenous steroids. Mild clinical improvement was recognized one month after treatment ended in one patient, but no change was seen in others. The possibility of a genetic disorder was considered only later since the diagnosis of ADEM was not convincing because of subacute presentation and slow improvement.

There was a marked imaging improvement in all individuals (Figure 1 and Figure 2). Mild brain atrophy was present in two patients (Table 2).

The mention of neuroradiological amelioration was associated with clinical improvement. All patients evolved with partial motor recovery over months and remained with residual motor disability, mostly lower limb spasticity, dysarthria, and, in two cases, mild dystonia. No intellectual disability was identified in six patients during follow-up. Patients 1 to 4 and 6 are attending regular school with dysarthria, spasticity, and some motor skill limitations, but with good communication and social abilities. They all learned how to read and write before 6 years old; without special education, there are no concerns regarding intellectual abilities. Patient 5 is currently 24 years old and finished traditional high school but developed severe and debilitating obsessive-compulsive (OCD) disorder in adolescence; his Mini-Mental State Examination is 30 (Table 1).

Patient 5 re-acquired all previous motor abilities (head control, sitting, walking); Patient 3 cannot sit without support. The other three patients re-acquired head control and sat without support but did not independently walk.

Those patients can be distinguished from typical ADEM presentation because of onset at a younger age, lack of consciousness impairment, and absence of fever or other inflammatory signs at disease onset. The subacute progression is also remarkable, with deterioration during a variable period of one to six months and the persistence of the motor disability after the subacute phase; none of those characteristics are expected for ADEM. Those, combined with the symptoms of relapse observed in a single patient, are the main findings oriented toward an investigation.

WES disclosed biallelic pathogenic variants in *RNASEH2A* (AGS4) or *RNASEH2B* (AGS2) in all patients:

1. Two individuals were homozygous for the variant p.Ala249Val in *RNASEH2A*. This rare missense substitution has never been reported in the literature at the time of the genetic test result, with a frequency at GnomAD [22] of 15 heterozygotes among 133,348 individuals (or ~1/9220). Both patients and their parents came from the same Northeastern Brazilian state of Ceara and are not known to be related. Alanine at codon 249 is highly conserved among biological species, and its substitution with valine is predicted to be deleterious by several computational programs (Polyphen, Provean, Mutation Taster).
2. Three individuals were homozygous, and one was compound heterozygous for the variant p. Ala177Thr in *RNASEH2B*, with an allele frequency according to GnomAD [22] of 0.001361 (377 heterozygous for this variant among 138,512 individuals, or 1/367). In homozygosity or compound heterozygosity, this variant has been reported several times in the literature and is responsible for 90% of pathogenic alleles found in AGS2. The other variant was a rare nonsense mutation (p.Arg58*) never before reported in the literature and present in heterozygosity in only 2 of 138,535 individuals (or approximately 1/70,000).
  Table 1 and Table 2 detail the six patients’ clinical, genetic, and radiological features.

## 4. Discussion

AGS is classically described as a progressive encephalopathy presenting in the first year of life with developmental delay, CSF pleocytosis and increased interferon-alpha levels, basal ganglia calcification, and white matter abnormalities. Furthermore, clinical heterogeneity and late-onset cases beginning in the second year of life have been previously reported [2,9,10,21]. Moreover, three previous case reports of AGS also described the improvement in white matter lesions [10,20,21]. Since the disease often initiates before myelination is completed, the naturally progressing myelination may improve the white matter abnormalities. However, normal myelination progress cannot fully explain the improvement in our cases once periventricular frontal white matter usually myelinates before subcortical white matter. For example, in Patient 4, the frontal periventricular hyperintensity was higher than the frontal-subcortical at 17 months (Figure 5C). It improved in a centripetal way at the age of 3 years (Figure 5E). That is not how normal myelination proceeds, except at terminal zones of myelination in peritrigonal regions.

Our patients share some clinical similarities to the previously reported cases with leukoencephalopathy recovery, as the onset of symptoms is in the first two years of life (ranging from 7 to 20 months in our series) after previous typical developmental milestones, a characteristic also presented in the reports of D’Arrigoo et al. [10] and La Piana et al. [21]. A possible immunologic trigger was present in all our patients: a respiratory infection for two subjects, an undetermined feverish condition for one, and a vaccination for three. The same group of researchers has also reported a temporal relationship to infection [10,21]. At the same time, a possible role of immunization trigger was pointed out by Orcesi et al. [9] in a late-onset AGS patient. Additionally, a subacute presentation was reported previously in late-onset AGS [10] and patients with radiological recovery [10,21], initially suggesting an acquired infectious/inflammatory disease.

Brain MRI features of five of the six patients shared similarities with ADEM, especially a patchy asymmetric distribution, which is uncommon in AGS [17] (Figure 1, Figure 2, Figure 3 and Figure 5). Diffuse homogeneous white matter lesions with brainstem involvement were seen in one patient with contrast enhancement, a feature, to our knowledge, never reported in AGS (Figure 4D,E). This patient with MRI signs of blood–brain barrier breakdown (patient 1) was the only one with a relapse four years after the first presentation, and he has the most pathogenic mutation. Some clinical characteristics in our series were distinct from ADEM, including onset at a younger age, subacute progression, and a lack of consciousness impairment (Figure 1).

The patchy distribution of the white matter lesions is not a common finding in AGS but has been described in a patient with *RNASEH2A* mutation [17]. The absence of severe atrophy or any identified atrophy in some patients is another peculiar finding in this group of patients [17].

The periventricular ependymal enhancement (Figure 4D) is uncommon in ADEM. This contrast enhancement pattern is seen mainly in autoimmune demyelination of optic neuromyelitis infectious ventriculitis caused by cytomegalovirus, lymphoma, or sarcoidosis [20,21]. Considering that all patients exhibited radiological and clinical improvement, even the ones who did not receive immunotherapy, we believe, similar to other colleagues, [20,21], that this is the natural course of this kind of AGS presentation and not the result of immunotherapy as previously suggested by D’Arrigoo et al. [10].

Brain calcification, leukoencephalopathy, and cerebral atrophy are the classic imaging hallmarks of AGS. Brain calcification was seen in only one of the six patients in a CT scan performed 17 days after disease onset (Figure 5A). In one patient, the image was performed 2 months after the acute decline, maybe too soon to see calcifications in case this was the evolution. However, in four of six patients, the CT scan was performed at least four years after the acute decline, and no calcifications resulting from the acute event were identified. We could better delineate the clinical course of patients with this peculiar presentation, identifying a subacute course of clinical symptoms, ranging from one to six months to reach the worst neurological condition, with loss of head control, spastic diplegia, and speech loss. Time to clinical improvement ranged from a few weeks to one year, so four patients recovered the ability to walk with support and speak, although dysarthria was present for five. No cognition issues were observed for all our subjects, a characteristic also described by D’Arrigoo et al. [10] and La Piana et al. [21]. We also had the same finding of the colleagues regarding no development of microcephaly. It also deserves to mention that our patients had, on average, seven years of follow-up, much longer than the previously reported cases. As seen in patient 1, demyelination relapse has not been previously reported in AGS.

WES disclosed biallelic mutations in RNASEH2A (two patients) and RNASEH2B (four patients). Variant detected in RNASEH2A (p.Ala249Val) was novel at the time of the genetic test result, but mutation seen in 7 of 8 pathogenic alleles of RNASEH2B (p.Ala177Thr) have been previously reported many times in patients with classical AGS2, being responsible for most of the pathogenic alleles in this condition [23]. Previous literature report that RNASEH2B-related disease could be associated with a milder phenotype (preserved cognitive function) [23,24].

All patients exhibited radiological and clinical improvement, even those who did not receive immunotherapy. We believe this is the natural course of disease [21,25] and not the result of immunotherapy, as previously suggested by the classical definition of AGS as a progressive and irreversible condition is challenged by those [10].

In the follow-up, variable degrees of spasticity and dysarthria were present and, without cognitive impairment, whenever this evaluation was possible. One patient developed an obsessive-compulsive disorder during adolescence. This outcome is not expected in classical AGS.

To our knowledge, few patients with a similar AGS presentation have been published previously [9,20,21]. No genetic study or clinical follow-up was presented in one patient [20], and no radiological follow-up was reported in another [9]. One patient was homozygous for the same *RNASEH2B* p.Ala177Thr [9] seen in three of our patients, and the other one is compound is heterozygous for it (p.Ala177Thr/p.Leu138Phe).

Genetically determined leukodystrophies usually have a relentlessly progressive course, even though some clinical improvement might occur, although not always accompanied by MRI improvement. Examples of partially reversible MRI changes have been reported in individuals with a biallelic mutation in *EARS2*, *HEPACAM, SLC13A3*, and, more recently, in *FDX2* [26]. On the other hand, acute inflammatory WM lesions, as in ADEM, characteristically improve or disappear [27].

Our report confirms the previous isolated case reports descriptions of the later onset phenotype of AGS and presents the most extensive clinical series of an atypical presentation of subacute clinical onset after an immunological trigger leading to regression of developmental milestones. After a variable period, partial clinical recovery occurred, accompanied by improved WM abnormalities. This paper highlights the possibility of clinical and radiological recovery in AGS being more common than initially described.

We suggest that AGS should be considered in the differential diagnosis of patients with a subacute presentation mimicking an acquired infectious/inflammatory disease, with atypical white matter involvement, even with normal CSF and without brain calcifications. So, one might be aware that AGS might be TORCH like and ADEM like. We highlight the importance of genetic techniques to the differential diagnosis of diseases and expand the phenotype of previously known hereditary conditions. The possibility of clinical and radiological spontaneous improvement might also be relevant for forthcoming clinical trials for AGS.

## Figures and Tables

**Table 1:** AGS partially reversible leukodystrophy: demographic data, clinical and laboratory features, and follow-up. OCD: obsessive-compulsive disorder. CSF: cerebral spinal fluid. All CSF cells were lymphocytes and monocytes. Coding sequence coordinators: Homo sapiens (human) genome assembly GRCh37 (hg19).

Patient	1	2	3	4	5	6
Age at onset	1 y 8 m Relapse at 5 y 8 m	1 y 2 mo	1 y 4 mo	1 y 5 mo	7 mo	1 y 4 mo
Developmental condition before the onset	Walk without support Says several words	Walk without support Says at least 3 words	Walk without support Says at least 5 words	Walk without support Says at least 5 words	Sit without support	Walk without support Says at least 5 words
Age at first evaluation	6 y	9 y 2 mo	8 y	5 y	21 y	2 y 1 mo
Last clinical evaluation	11 y	14 y	12 y	9 y	24 y	6 y
Current age	11 y	14 y	12 y	10 y	25 y	6 y
Sex	M	F	M	M	M	M
Previous infection/immunization	Respiratory infection	Relapse-remitting fever	Multiple immunizations ^(PCV−10, TDp, Hib, and OPV)^ 10 days before the onset	Yellow fever vaccine, 7 days before the onset	Respiratory infection	Multiple immunizations ^(MMR, varicella, TDp, and OPV)^ 4 days before the onset
Consanguinity	No	Parents are first cousin	No	No	No	No
First clinical features	Ataxia Drowsiness Motor regression Spastic diplegia	Motor and language regression Spastic diplegia	Motor and language regression Spastic diplegia	Motor and language regression Spastic diplegia	Strabismus Seizure Motor regression Hypotonia	Motor and language regression Spastic diplegia
Time to reach the nadir of disease	1st event: 1 mo 2nd event: a few days	2 mo	6 mo	1 mo	3 mo	2 mo
Clinical features during the nadir of disease	Loss of head control Spastic diplegia	Loss of head control Speech loss Spastic diplegia	Loss of head control Speech loss Spastic diplegia Dystonia	Loss of head control Speech loss Spastic diplegia	Loss of head and sitting control	Loss of head control Speech loss Spastic diplegia
Treatment in the acute phase	Immunoglobulin in the 1st event					
	IV steroid in both events	IV steroid	None	IV steroid	None	IV steroid
Time to start milestones recovery	3 mo in both	1 y	1 y	1 y	6 mo	1 m
Actual clinical follow-up	Spastic diplegia Dysarthria Walk with support No concerns regarding cognition	Spastic diplegia Dysarthria Walk with support No concerns regarding cognition	Spastic diplegia Dysarthria Sit without support Dystonia No concerns regarding cognition	Spastic diplegia Dysarthria Walk with support No concerns regarding cognition	Spasticity of the right arm and left leg OCD Walk without support No concerns regarding cognition	Spastic diplegia Dysarthria Sit without support
CSF	21 cells/mm^3^: 1st event 10 cells/mm^3^: 2nd event	5 cells/mm^3^	8 cells/mm^3^	2 cells/mm^3^	4 cells/mm^3^	5 cells/mm^3^
Molecular analysis	AGS2 *RNASEH2B* p.Gln58*/ p.Ala177Thr	AGS2 *RNASEH2B* p.Ala177Th/p.Ala177Thr	AGS4 *RNASEH2A* p.Ala249Val/p.Ala249Val	AGS2 *RNASEH2B* p.Ala177Thr/p.Ala177Thr	AGS4 *RNASEH2A* p.Ala249Va/ p.Ala249Val	AGS2 *RNASEH2B* p.Ala177Thr/p.Ala177Thr
GRCh37 (hg19)	chr13:51.503.646/ chr13:51.519.581	chr13:51.519.581	chr19:12.924.005	chr13:51.519.581	chr19:12.924.005	chr13:51.519.581

**Table 2:** AGS partially reversible leukodystrophy. Acute phase and follow-up MRI and CT scan. Y: years. Mo: months.

Image Modality	Patient	1	2	3	4	5	6
	**Distribution**	Diffuse/homogeneous	Patchy/asymmetric	Patchy/asymmetric	Patchy/asymmetric	Patchy/asymmetric	Patchy/asymmetric
**MRI at onset**	**Predominant location**	Diffuse	Frontal and anterior temporal	Frontal and anterior temporal	Frontal and anterior temporal	Frontal and anterior temporal	Frontal and parietal
	**Infratentorial involvement**	Pons	-	-	-	-	Pons
	**Contrast enhancement**	+	-	-	-	-	-
**MRI at follow up**	**Months after onset**	1st episode 8 mo (2 y 3 mo old)					
		2nd episode					
		12 mo	4 y	10 mo	24 mo	13 y	5 mo
		(6 years old)	(6 years old)	(2 y 2 mo old)	(3 y 2 mo old)	(13 years old)	(20 mo old)
	**WM lesions improvement**	Marked	Marked	Marked	Marked	Marked	Marked
	**Brain** **Atrophy**	Mild	-	Mild	-	-	-
**CT scan**	**Time after** **onset**	5 y	4 y	7 y	1 mo	13 y	2 mo
	**Calcification**	-	-	-	Basal ganglia	-	-

## Data Availability

The data presented in this study are available on request from the corresponding author. The data are not publicly available due to personal identifying information.
